# Are Mechanical Turk worker samples representative of health status and health behaviors in the U.S.?

**DOI:** 10.1371/journal.pone.0198835

**Published:** 2018-06-07

**Authors:** Kelly Walters, Dimitri A. Christakis, Davene R. Wright

**Affiliations:** 1 Center for Child Health, Behavior, and Development, Seattle Children’s Research Institute, Seattle, WA, United States of America; 2 Department of Pediatrics, University of Washington School of Medicine, Seattle, WA, United States of America; East Tennessee State University, UNITED STATES

## Abstract

**Introduction:**

Amazon’s Mechanical Turk (MTurk) is frequently used to administer health-related surveys and experiments at a low cost, but little is known about its representativeness with regards to health status and behaviors.

**Methods:**

A cross-sectional survey comprised of questions from the nationally-representative 2014 Behavioral Risk Factor Surveillance System (BRFSS) and 2014 National Health and Nutrition Examination Survey (NHANES) was administered to 591 MTurk workers and 393 masters in 2016. Health status (asthma, depression, BMI, and general health), health behaviors (influenza vaccination, health insurance, smoking, and physical activity), and demographic characteristics of the two MTurk populations (workers and masters) were compared to each other and, using Poisson regression, to a nationally-representative BRFSS and NHANES samples.

**Results:**

Workers and master demographics were similar. MTurk users were more likely to be aged under 50 years compared to the national sample (86% vs. 55%) and more likely to complete a college degree than the national sample (50% vs. 26%). Adjusting for covariates, MTurk users were less likely to be vaccinated for influenza, to smoke, to have asthma, to self-report being in excellent or very good health, to exercise, and have health insurance but over twice as likely to screen positive for depression relative to a national sample. Results were fairly consistent among different age groups.

**Conclusions:**

MTurk workers are not a generalizable population with regards to health status and behaviors; deviations did not follow a trend. Appropriate health-related uses for MTurk and ways to improve upon the generalizability of MTurk health studies are proposed.

## Introduction

Mechanical Turk is a crowdsourcing platform developed by Amazon through which, broadly speaking, “requestors” may hire “workers” to complete “human intelligence tasks” for a small cost [1]. In this case, crowdsourcing refers to accomplishing a task by opening it up to the public. Given the diversity of the worker sample, large number of workers, quick turnaround time and low cost of work, an increasing number of researchers have identified Mechanical Turk (MTurk) workers as an effective participant pool for surveys. Evidence from behavioral science experiments conducted on MTurk suggests that workers can produce results that are just as valid and reliable as field and laboratory experiments.[2, 3] Quality is moderated by the requester; after submitting a task (e.g., a survey), requestors may approve or reject the work completed by the worker based on its quality, and Amazon tracks workers’ rejection rates, providing motivation and accountability for workers to perform their tasks with diligence.

Use of MTurk as a research tool has been increasing rapidly. In 2011, just 61 studies using MTurk had been published; 1200 studies were published by 2015.[4] A central concern surrounding using Mechanical Turk for research is the generalizability of workers to a broader population. A growing body of research has examined demographic and political characteristics of MTurk workers.[5–8] Research assessing characteristics of MTurk workers as social science research participants concludes that workers represent a large and diverse population, yet MTurk workers are a convenience sample.[7]

Beyond social, behavioral and political science research, crowdsourcing has been used to identify a participant pool for a number of health and medical studies in which researchers engage workers in tasks including health surveillance (i.e. reporting disease symptoms) and completion of health surveys.[8] While some studies use MTurk as a way to assist in survey development or pilot new health measures, a number of studies appear to treat MTurk workers as being representative of the general public or all persons with a particular health condition, failing to acknowledge differences in health characteristics between their convenience sample and national averages. The representativeness of an MTurk sample with regards to Americans’ health behaviors, access to health care, and physical and mental health status is not fully understood. Even if MTurk workers represent a demographically diverse sample, if their health characteristics are not representative of the broader U.S. population, MTurk surveys may have systemic biases and limited external validity. One study has previously assessed health characteristics within MTurk, but focuses on cardiovascular-related health status more so than general health behaviors and characteristics.[9] The aim of this study was to compare a variety of health characteristics within a U.S. MTurk sample to those from national health surveys.

## Methods

### Study population

A survey was administered to two groups: regular MTurk workers (“workers”) and “masters”, a subset of experienced workers who have demonstrated accuracy for certain MTurk tasks, validated by previous MTurk requesters. The surveys were programmed in SurveyMonkey, and administered through Amazon’s Mechanical Turk platform. Potential respondents who logged into the MTurk platform during the week in which the survey was administered were informed that they had an opportunity to take a survey about “self-reported health of people in the United States,” that the survey would be less than 10 minutes long, and that they must have an U.S.-based IP address in order to be qualified for the survey. Surveys were launched daily at 2 pm Eastern Standard Time over a one-week period in 2016 in order to allow individuals from all time zones to complete the survey during the work day. Workers were offered $0.25 to complete the survey, and masters were offered $1.00.

### Survey design

The survey was designed to allow for the direct comparison of health characteristics of MTurk workers to the national population. A review of published MTurk surveys was used to determine an adequate sample size. Demographic questions and questions chosen to capture the scope of health behaviors, health access, and physical and mental health status were taken directly from two probability-based national surveys: the Behavioral Risk Factor Surveillance System (BRFSS), and the National Health and Nutrition Examination Survey (NHANES). Depressive symptoms were measured using the two-item Patient Health Questionnaire (PHQ-2), a validated and frequently used metric with 97 percent sensitivity and 67 percent specificity for detecting major depression among adults.[10] Two attention check questions (e.g. “What was this survey about?”) were added to ensure thoughtful completion of the survey.

### Statistical analysis

Workers who didn’t complete all survey questions and workers who failed either attention check were dropped from the sample. For survey completers, workers’ versus masters’ responses were compared using a Pearson’s chi-squared test. MTurk versus national demographic information was summarized descriptively using frequency tables. Welch’s t-test accounting for unequal variances and the Kolmogorov-Smirnov test for distributions were used to compare continuous variables.

Respondents were asked to report their height and weight in feet and inches and pounds, respectively; body mass index (kg/m^2^) was calculated in accordance with CDC guidelines.[11] The PHQ-2 questions were scored in accordance with published guidelines, ranging from 0–6, with a score ≥4 used as the optimal cut point for depression screening.[12]

Differences in health characteristics between the MTurk and national samples were modeled using a Poisson regression for each characteristic. Poisson regression was chosen because it can provide more accurate and interpretable estimates than logistic regression.[13] Regression analyses were conducted overall and stratified by age. Regression analyses were adjusted for sex, race, level of education, income, and current working status. An a priori α = 0.05 was used as a cutoff for model inclusion and to assess statistical significance. The svy package in Stata (version 14) was used to adjust for the complex sampling design in BRFSS and NHANES.[14] BRFSS data from all 50 states was used in the analysis. The BRFSS sample design variables used were STSTR (Sample Design Stratification Variable), _PSU (Primary Sampling Unit), and _LLCPWT (Final Weight: Landline and Cell­Phone Data). NHANES was used as the nationally representative sample for depression data (i.e. the PHQ-2), and the BRFSS was used for the remainder of the survey questions. Given the entirely online nature of the study, MTurk survey participants did not provide written consent, but were shown consent language and digitally agreed to participate in the research study. This study was approved by the Seattle Children’s Institutional Review Board.

Primary data collected as part of this study are publicly available at the Harvard Dataverse (DOI: 10.7910/DVN/I7U3GT).

## Results

In total, 1,086 surveys were initiated, and 102 were dropped from analysis due to duplicate IP addresses (n = 25), lack of completion of the survey (n = 65) or failing to correctly pass the attention check (n = 12), leaving 984 individual respondents. There were no statistically significant differences detected between workers (n = 591) and masters (n = 393), except with respect to gender (workers = 41% female, masters = 48% female, p = 0.046). Thus, all subsequent analyses were performed grouping workers and masters together, hereafter collectively known as Turkers. The geographic distribution of respondents was similar to that of the US population, with California, Texas, Illinois, Florida, North Carolina and New York having the highest proportions of respondents.

Compared to the national sample, Turkers had a lower proportion of female respondents (44.4 vs. 51.4%), were more likely to be currently employed (78.2% vs. 49.4%), were less likely to earn more than $75,000 (18.4 vs. 25.6%), were more likely to be of White (79.3% vs. 72.1%) or Asian race (7.9% vs. 2.3%) and were more likely to have completed college (49.9% vs. 25.8%). (Table 1) Turkers were significantly younger than the national population by 19.9 years, (p<0.001). Turkers also had a lower BMI than the national sample, although this difference may not be clinically meaningful. (Table 2)

**Table 1:** Characteristics of Mechanical Turk workers versus national 2014 BRFSS sample.

	Mechanical Turk sample (%) (n = 984)	National sample (%) (n = 464,664)
	*Percent*	*Weighted percentage*
**Age**		
< 30 years	34.04	21.22
30–49 years	38.11	16.66
40–49 years	14.23	16.65
≥ 50 years	13.62	45.47
**Sex**		
Female	44.41	51.35
**Work**		
Currently employed *	78.15	49.39
Not currently employed ^†^	18.9	49.65
Other^‡^	2.95	0.96
**Income**		
Less than $35,000	41.46	35.71
$35,000 to less than $50,000	17.68	11.6
$50,000 to less than $75,000	20.02	12.56
$75,000 or more	18.39	25.59
Other^§^	2.43	14.46
**Race**		
White	79.27	72.1
Black or African American	5.79	12.64
Hispanic or Latino	5.28	8.57
Asian	7.93	2.25
Other^|^	1.73	4.45
**Education**		
Some high school or less	1.02	14.66
High school or GED	14.02	28.24
Some college	34.45	30.58
College graduate	49.90	25.79
Refused	0.61	0.73

* Includes those working for pay, with a job or business but not at work, or working, but not for pay at a family-owned job or business

^†^ Includes those who are looking for work, not working at a job or business and not looking for work, out of work, homemakers, retired, students, and those unable to work

^‡^Other includes those who refused to answer, “Not ascertained,” and “Don’t Know”

^§^ Other includes those who refused to answer, “Not sure,” and “Don’t Know”

^|^ Other includes “Native Hawaiian or Pacific Islander, American Indian or Alaskan Native, Some other group, Prefer not to answer, and Don’t Know/Not Sure

**Table 2:** Summary and comparison of continuous variables between MTurk and national samples.

Variable	MTurk mean	BRFFS mean	Difference	Welch’s p-value	Combined K-S	K-S p-value
Age	35.60	55.49	19.68	<0.001	0.55	<0.001
BMI (kg/m^2^)	26.73	27.89	1.16	<0.001	0.16	<0.001

Abbreviations

MTurk: Mechanical Turk

BMI: Body Mass Index

BRFSS: Behavioral Risk Factor Surveillance Survey

K-S: Kolmogorov-Smirnov

Table 3 shows the results of a series of multivariate Poisson regressions in which the health behavior and health status variables were the dependent variable, with sample type (MTurk or national) as the primary predictor. Results are presented overall and stratified by age group. Almost all differences between Turkers and the national sample were statistically significant. Turkers were less likely than the national sample to be vaccinated for influenza (IRR = 0.51, 95% CI: 0.51, 0.52); this disparity was more pronounced among 18 to 29 years olds than older age groups. Overall, Turkers were less likely than the national sample to smoke (IRR = 0.62, 95% CI: 0.61, 0.63), although this difference varied substantially by age group. Smoking behavior of older Turkers was more similar to that of their national peers than that of younger Turkers (IRR = 0.94, 95% CI: 0.92, 0.97). Turkers were less likely to have asthma than the national sample (IRR = 0.76, 95% CI: 0.75, 0.78), although 30–39 year olds were more similar to national counterparts than other age groups in this regard (IRR = 0.83, 95% CI: 0.79, 0.87). In contrast, however, Turkers were less likely to self-report being in excellent or very good health than the national sample (IRR = 0.67, 95% CI: 0.66, 0.67), and those aged < 50 were two to three times more likely to screen positive for depression than their national counterparts (IRR 2.39–3.29). Turkers ≥ 50 years were less likely than their national counterparts to screen positive for depression (IRR = 0.61, 95% CI: (0.38, 0.99). Lastly, Turkers were slightly less likely to have participated in physical activity (IRR = 0.96, 95% CI: 0.95, 0.96) or have health insurance (IRR = 0.88, 95% CI: 0.88, 0.88) relative to the national sample, although these relative risks are less strong than relative risks for other health characteristics.

**Table 3:** Adjusted incidence of health characteristics between MTurk and national samples, overall and stratified by age group.

	IRR (95% CI)				
Health characteristic	All ages	Aged 18–29 years	Aged 30–39 years	Aged 40–49 years	Aged ≥50 years
Influenza vaccination	0.51 (0.51, 0.52)	0.48 (0.46, 0.50)	0.63 (0.61, 0.64)	0.65 (0.63, 0.67)	0.64 (0.63, 0.64)
Smoking*	0.62 (0.61, 0.63)	0.34 (0.33, 0.36)	0.52 (0.51, 0.54)	0.61 (0.59, 0.63)	0.94 (0.92, 0.97)
Physical activity^†^	0.96 (0.95, 0.96)	0.90 (0.89, 0.91)	0.91 (0.90, 0.92)	0.97 (0.95, 0.98)	1.02 (1.02, 1.03)
Health insurance	0.88 (0.88, 0.88)	0.93 (0.92, 0.94)	0.93 (0.92, 0.94)	0.86 (0.85, 0.87)	0.82 (0.82, 0.83)
Asthma	0.76 (0.75, 0.78)	0.54 (0.52 0.57)	0.83 (0.79, 0.87)	0.61 (0.57, 0.64)	0.71 (0.69, 0.73)
General health^‡^	0.67 (0.66, 0.67)	0.59 (0.58, 0.60)	0.62 (0.61, 0.63)	0.59 (0.58, 0.60)	0.81 (0.80, 0.81)
Depression^§^	2.26 (1.68, 3.02)	2.39 (1.13, 5.03)	3.17 (1.95, 5.13)	3.29 (2.06, 5.26)	0.61 (0.38, 0.99)

Note: Poisson regression models used to generate incidence rate ratios (IRR) adjusted for sex, race, level of education, income, and current working status. All figures represent weighted estimates.

* Smoking cigarettes every day or some days compared to not at all

^†^ Participating in any physical activities or exercises such as running, calisthenics, gardening or walking for exercise in the past month

^‡^ Excellent or very good self-reported health compared to good, fair, or poor self-reported health

^§^ PHQ-2 score ≥4 compared to PHQ-2 score < 4

## Discussion

Because Mechanical Turk has been shown to produce data quickly and at a low cost, it has been used extensively to conduct health research. [15–26] It is therefore important to understand any biases within and the external validity of this population. This study found that Turkers were generally younger, of lower socioeconomic status, and less racially/ethnically diverse than the national population. These demographics are consistent with other recent studies examining Turkers crowdsourced as a study population.[3, 6, 27] But in this sample, Turkers differed significantly from nationally representative samples in almost every health-related variable that was measured, even after controlling for demographic covariates. Even if sample weights could be employed to make the demographics representative, Turkers’ health behaviors are not representative of the national population for the purposes of health research, independent of demographic differences, and MTurk surveys should be clear that findings are not generalizable. Most notable was the large difference in depressive symptoms between the two groups—Turkers were more than twice as likely to exhibit depressive symptoms than the national sample; relative risks were >3 among 30–49 year olds. This is consistent with previous findings that Turkers are more likely to experience anxiety or depression than other traditional community or epidemiological samples.[28] Researchers will therefore find a readily available population for depression-related health tasks, but publications should report on other sample health characteristics so readers can assess potential biases in data.

Apart from slight differences in gender balance between the groups, there were no significant differences in respondents between the MTurk workers and MTurk masters surveyed. This suggests that the worker and master samples are comparable with regards to demographics and health characteristics, and researchers may be better off using workers instead of masters for survey-related tasks as they represent a larger sample and a more cost-effective means of obtaining similar data. However, questions asked in the surveys were simple and not cognitively demanding; differences between these groups could emerge if more arduous tasks were to be administered.

Interestingly, differences between the MTurk and national samples did not appear to follow any trends. For example, Turkers were less likely to engage in healthy behaviors such as getting vaccinated for influenza or engaging in physical activity, yet were also less likely to engage in the unhealthy behavior of smoking. Compared to a national sample, Turkers were less likely to have asthma (a “healthier” characteristic), but also reported lower general health status. This suggests that these populations may also differ for a number of unobservable health characteristics, and may do so in unpredictable patterns.

One previous study by Yank et al. examined demographic and health characteristics in the MTurk population versus a national sample, with similar findings to ours regarding age, gender, and race.[9] While both studies used different methods (Poisson vs. logistic regression) and the relative risks we report and the odds ratios they report are not directly comparable, it appears that Yank et al. found greater disparities in physical activity between MTurk and national samples compared to our analysis, especially among older adults. However, we assessed any physical activity in the last month whereas Yank et al. assessed the frequency, duration, and level of vigor of physical activity. Unlike the present analysis, they did not find differences in smoking behaviors between samples among those aged ≥30, although they examined current and former smoking status whereas the present study assessed current smoking status only. Yank et al. measured a wider range of cardiovascular-related chronic conditions, including diabetes, hypertension, and hyperlipidemia, but found few significant differences in these conditions among age groups, especially among those aged ≥ 50. Yank et al. conclude 40 to 59 year old Turkers were most representative with respect to smoking, diabetes, hypertension, and hyperlipidemia status, whereas large disparities (i.e. relative risk ~ 0.60) in a wider range of health conditions and health behaviors were observed in this subsample, especially among 40 to 49 year olds.[9]

There are several possible explanations for the differences in findings presented in this study and those from Yank et al. First, the MTurk worker population has high turnover. Almost a quarter of MTurk workers retire and are replaced every quarter.[29] The study by Yank et al. was administered in the summer of 2015 whereas the present study was administered in February 2016, so the two studies could have examined different samples. Additionally, the present survey was launched during the work day whereas Yank et al. fielded their survey at different times over a 6-week period.[9]

Future researchers sampling from Mturk for health related studies in hopes of generating generalizable data should make sure to address the differences found between these groups in their research design and analysis. In this paper, we have surveyed and published differences that we believe to be of interest to researchers in the medical and public health fields. Depending on the research question and aims of any given study using Mturk workers as a sample, these differences may or may not be salient. We hope that our findings can be used as a helpful guide for future researchers to use upon drafting their Mturk populations. It is, of course, the responsibility of these researchers to take these differences into account and use the necessary tactics, such as pre-screening survey questions or appropriately weighting samples, to address them. This study has also reiterated that demographically similar populations may still differ with regards to health behaviors, and so researchers using pre-screening questions will need to make sure to screen for both demographics and behavioral characteristics to ensure a diverse, representative sample.

Academic researchers have expressed apprehensions about using MTurk as a convenience sample outside of concerns of representativeness. Some are uncomfortable with the idea of commercial dependence on a service that could be removed from Amazon at any time.[4] Moreover, while characterized as anonymous, recent research reveals that workers’ identifying information can be readily assessed via workers’ IDs, raising concerns about the collection of Protected Health Information and increased institutional review board scrutiny.[30] Finally, others have expressed ethical concerns about low participant pay, which often falls below the 2016 federal minimum wage of $7.25 per hour, or $0.12 per minute.[4] On one hand, paying especially low rates may be taking advantage of a vulnerable population. On the other hand, paying high rates may be considered coercive. While a number of Turkers want to contribute to research, others are seeking a paycheck, routinely posting links to short, high-paying tasks on message boards. Either approach, underpaying or grossly overpaying relative to standard MTurk rates, could lead to a biased sample—solely inexperienced workers willing to accept lower-paying tasks or experienced workers seeking out high paying tasks.

MTurk could still be a valuable source of data for health-related tasks that involve problem-solving, data processing, and pilot surveys, which would not necessarily require a generalizable population.[8] MTurk has also shown promise as a helpful tool for health researchers outside of its use as a subject pool. Turkers have been used to aid in content analysis psychological research with adequate reliability and validity [31], and MTurk has been used to pilot and design clinical tools such as Visualizing Health, a health-related data visualization tool developed to portray health data to general audiences.[32]

### Limitations

This study had some limitations. The survey was launched during the day and throughout a one-week window, which may limit the representativeness of the sample compared to all MTurk workers. However, the survey was administered in this way in order to make it available during a similar time period in all US time zones. Having the survey available during the day likely limited the demographics of our survey population. Future research should consider fielding the survey at different times throughout the day to recruit a more diverse sample of Mturk workers. Mturk requesters now have the option to stagger survey launch times, thus administering future surveys at a range of time points can be accomplished with minimal effort.

All responses were self-reported, although this limitation exists for many of the questions in the parent survey. The hourly rate for survey completions was low, which may have also influenced the type of respondents and quality of response. However, this limitation is not unique to this study and would apply to most brief MTurk surveys.[3]

There was a time discrepancy between BRFSS and NHANES data, which was collected in 2014, and the Mturk survey was launched in 2016. This could account for differences between the groups, however this was the most recent data set available at the time and thus represents the closest measurable approximation for a nationally representative sample.

Lastly, this survey included a limited number of questions to assess health characteristics. This was done to limit the survey length and respondent burden, and we intentionally chose to address well-known and prevalent issues such as receipt of influenza vaccines, depressive symptoms, and asthma to increase the prevalence in our relatively small sample.

## Conclusions

While MTurk may be an expedient means to recruit survey respondents, its workers are not a generalizable population with regards to health status and health behaviors. Sample weights may need to be employed in data analysis of MTurk surveys to ensure representativeness, but even if demographic representativeness is achieved, Turkers’ health behaviors and health status may not be representative of the U.S. population as measured by large national health surveillance surveys. In particular, our findings raise questions about the validity of MTurk surveys that relate to health conditions that affect older populations, which are not prevalent among MTurk workers.
